# Questioning the Rhythm: A Closer Look at Heart Rate Trends in Atrial Fibrillation

**DOI:** 10.1002/clc.70150

**Published:** 2025-05-13

**Authors:** Ibrahim Nagmeldin Hassan

**Affiliations:** ^1^ Faculty of Medicine University of Khartoum Khartoum Sudan

**Keywords:** adverse outcomes, atrial fibrillation, heart rate variability, post hoc analysis, rate control

## Conflicts of Interest

The author declares no conflicts of interest.

Dear Editor,

Kodani et al.'s recent post hoc analysis of the J‐RHYTHM Registry adds an important perspective to heart rate (HR) management in non‐valvular atrial fibrillation (NVAF), suggesting that both excessive increases and consistently high HR are associated with adverse outcomes [1]. While the findings are provocative, they rest on several methodological and interpretive weaknesses that warrant a more cautious interpretation.

First, the use of only two time points—baseline HR and the final HR before an event or last follow‐up—as the basis for trend analysis is an oversimplification. Atrial fibrillation is inherently dynamic, and HR can fluctuate markedly with physical activity, stress, or medication changes. Capturing HR trends through just two snapshots neglects the nuances of temporal variation and patient trajectories, potentially masking critical intermediate changes or transient arrhythmias that may carry their own prognostic weight.

Second, the study does not adequately account for medication titration, adherence, or adjustments during the 2‐year follow‐up. Patients with rising HRs may have experienced worsening clinical status, necessitating reduced beta‐blocker doses or shifts to rhythm‐control strategies. Without addressing these changes, it's difficult to determine whether HR elevation is a causative factor or merely a marker of underlying decompensation [2].

Moreover, stratifying patients into rigid HR quartiles introduces arbitrariness. A uniform cut‐off of ≥ 80 bpm may not carry the same prognostic significance across age groups or comorbidity profiles. Such one‐size‐fits‐all stratification may oversimplify risk and obscure clinically relevant thresholds [3]. Additionally, although the authors suggest a benefit from modest HR reductions, several hazard ratios carry wide confidence intervals that approach non‐significance—raising concerns about multiple comparisons and type I error.

Crucially, as an observational study, causal inference is inherently limited. The observed associations may reflect reverse causality—where increased HR signals frailty, subclinical illness, or impending adverse events—rather than modifiable targets. Prior trials, such as RACE II, failed to show superiority of strict over lenient rate control for major outcomes, reinforcing the importance of individualized therapy based on symptoms and clinical context [4].

Future studies should employ continuous monitoring or longitudinal modeling to better characterize HR variability and its prognostic value. Until then, clinicians should interpret these findings as hypothesis‐generating rather than practice‐changing.

## Author Contributions

Ibrahim Nagmeldin Hassan conceptualized the idea. Ibrahim Nagmeldin Hassan wrote the main manuscript text.

## Data Availability

The author has nothing to report.
